# Transcranial doppler ultrasonography should it be the first choice for persistent foramen ovale screening?

**DOI:** 10.1186/1476-7120-12-16

**Published:** 2014-05-22

**Authors:** Monika Komar, Maria Olszowska, Tadeusz Przewłocki, Jakub Podolec, Jakub Stępniewski, Bartosz Sobień, Rafał Badacz, Anna Kabłak-Ziembicka, Lidia Tomkiewicz-Pająk, Piotr Podolec

**Affiliations:** 1 Department of Cardiac and Vascular Diseases, John Paul II Hospital, Institute of Cardiology, Collegium Medicum, Jagiellonian University, Krakow, Poland; 2 Department of Interventional Cardiology, John Paul II Hospital, Institute of Cardiology, Jagiellonian University Medical College, Krakow, Poland

**Keywords:** Persistent foramen ovale, Transcranial color doppler ultrasound, Transesophageal echocardiography

## Abstract

**Background:**

Persistent foramen ovale (PFO) is considered a cause of cryptogenic stroke and a risk factor for neurological events in young patients. The reference standard for identifying a PFO is contrast-enhanced transesophageal echocardiography (TEE).

The goal of this study was to evaluate the feasibility of transcranial color Doppler (TCD) and its diagnostic sensitivity compared with TEE.

**Methods:**

We investigated 420 patients admitted to our department with cryptogenic stroke, transient ischemic attacks or other neurological symptoms. All patients underwent TCD and TEE evaluation. TCD and TEE examinations were performed according to a standardized procedure: air-mixed saline was injected into the right antecubital vein three times, while the Doppler signal was recorded during the Valsalva maneuver. During TCD the passage of contrast into the right-middle cerebral artery was recorded 25 seconds following the Valsalva maneuver.

**Results:**

We detected a right-to-left shunt in 220 patients (52.3%) and no-shunts in 159 patients (37.9%) with both TCD and TEE. In 20 (4.8%) patients TEE did not reveal contrast passage which was then detected by TCD. In 21 (5.0%) patients only TEE revealed a PFO. The feasibility of both methods was 100%. TCD had a sensitivity of 95% and a specificity of 92% in the diagnosis of PFO.

**Conclusions:**

TCD has a relatively good sensitivity and specificity. TCD and TEE are complementary diagnostic tests for PFO, but TCD should be recommended as the first choice for screening because of its simplicity, non-invasive character, low cost and high feasibility.

## Background

Persistent foramen ovale (PFO) is considered a cause of cryptogenic stroke and a risk factor for neurological events in patients under 40 years of age. PFO has been also associated with several disease processes such as arterial gas embolism due to decompression, or platypnea-orthodeoxia syndrome [1-4]. Although the clinical significance of PFO in relation to cryptogenic stroke is still debated, identification and assessment of this abnormality are nowadays a routine diagnostic procedure.

Agitated saline serum has been used in transthoracic echocardiography (TTE), transesophageal echocardiography (TEE) and transcranial color Doppler ultrasound (TCD) for the detection of intracardiac shunts. The reference standard for identifying a PFO is contrast-enhanced TEE but it is a semi-invasive, inconvenient and stressful for the patients and also quite expensive procedure.

Some studies have analyzed the most suitable strategy for the screening, diagnosis and quantification of PFO [5-8]. The aim of this study was to evaluate the feasibility of transcranial Doppler and its diagnostic sensitivity compared with transesophageal echocardiography.

## Methods

### Patient population

We investigated 420 consecutive patients [(260 females, 160 males); mean age 34.8 ± 16.7 (13, 65) years] admitted to our department with cryptogenic stroke, transient ischaemic attacks (TIA) or other neurological symptoms between 2007 and 2014. Most patients (over 50%) had a history of TIA. The main demographic characteristics of the patients are summarized in Table 1.

**Table 1:** Demographic characteristics of the patients

	**Patients (n = 420)**
Smoking	205 (48.8%)
Obesity	72 (17.4%)
Diabetes mellitus	19 (4.5%)
Hypertension	64 (15.2%)
Dyslipidemia	29 (6.9%)

### Protocol

All patients underwent TCD and TEE evaluation. The studies were performed on the same day by two different experienced cardiologists, unaware with respect to the result of the other study. The patients were examined in the fasting state. Written informed consent was obtained from all patients. The patients were in left lateral decubitus position during both studies. The studies were performed at resting state and during the Valsalva maneuver after contrast injection. The patients had been trained in performing the Valsalva maneuver before study. The training phase included up to 5 Valsalva maneuver attempts, with at least two resulting in septal shifting or reduced middle cerebral artery flow velocity which was followed by contrast injection and the Valsalva maneuver solicited. The study was repeated at least three times.

### Agitated saline contrast test

TCD and TEE examinations were performed according to a standardized procedure. In brief, 10 mL of air-mixed saline was injected into the right antecubital vein at three different times, while the Doppler signal was recorded during the Valsalva maneuver. During TCD the passage of contrast into the right-middle cerebral artery was recorded 25 seconds after the Valsalva maneuver. A mixture of 9 mL physiological saline and 1 mL air was agitated 10 times in 2 10-mL syringes connected to a 3-way stopcock to exchange the air-saline mix and achieve good dilution. The bolus of saline solution was prepared and injected by the same nurse using the same method in all studies. The patients started the maneuver about 5 seconds after contrast injection, pressing against the closed glottis for at least 10 seconds. They then performed a deep expiration and inspiration, followed by a deep expiration.

### Transcranial doppler ultrasonography

**Figure 1:**
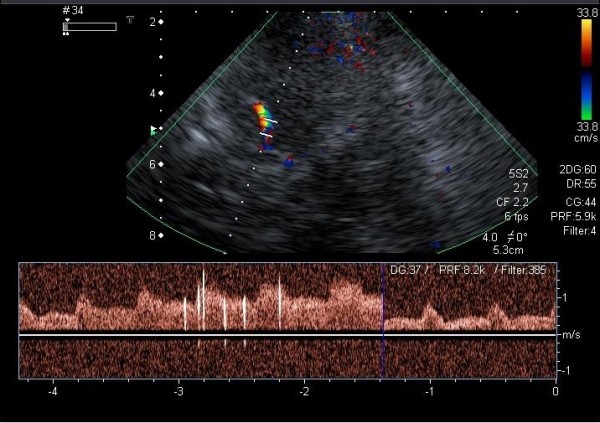
**Contrast TCD during the Valsalva maneuver.** The middle cerebral artery was identified with color Doppler. The passage of contrast into the right middle cerebral artery (MCA).

A baseline TCD examination was performed with a Toshiba Power Vision echo-machine using a 2-MHz probe, according to standard practice guidelines. Middle cerebral artery flow was monitored through the temporal bone window. The middle cerebral artery was identified with color Doppler in its proximal portion and insonated bilaterally (Figure 1). On TCD study, the effectiveness of the Valsalva maneuver was verified by a reduction of the middle cerebral artery flow velocity, in comparison with the basal spectrum. TCD was considered positive if at least one microembolic signal was recorded on TCD spectrum within 25 seconds from contrast injection. The shunt was defined as small (1, 10 microembolic signals), medium (>10 microembolic signals), or large (>10 microembolic signals with “curtain”).

### Transesophageal echocardiography

**Figure 2:**
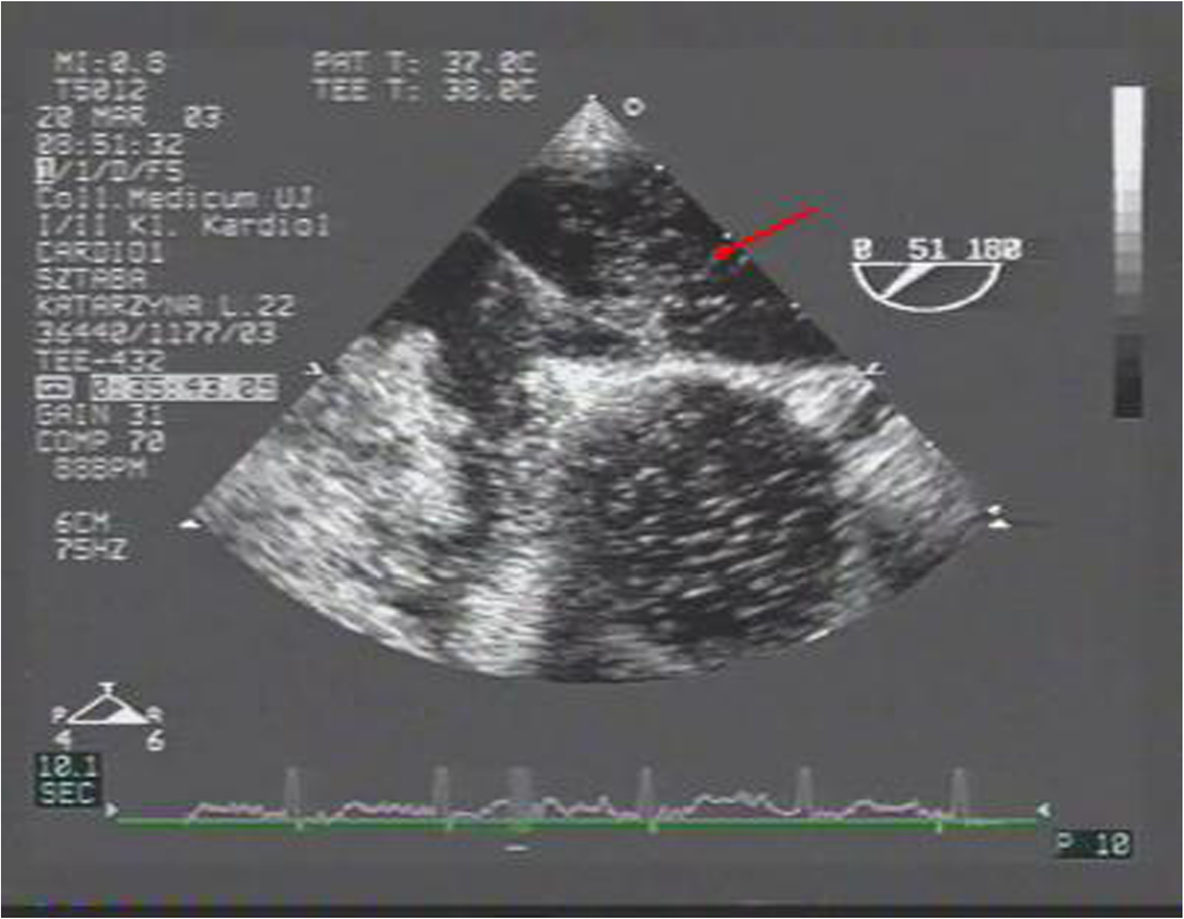
**Contrast TEE during the Valsalva maneuver.** Contrast agent in the left atrium.

The patients received local pharyngeal anesthesia with 10% topical lidocaine for TEE study. A TEE study was performed using a Toshiba Power Vision machine with a 5.0-MHz multiplane probe, according to a standard protocol including color flow Doppler data. The atrial septum was analyzed from the transverse mid-esophageal four-chamber view to the longitudinal biatrial-bicaval view (Figure 2). Since PFO was not clearly recognized by a bicaval view at 90° rotation, additional image planes (60°, 90° and/or 110°-130°) were used to better analyze the atrial septum. On TEE study, the effectiveness of the Valsalva maneuver was verified by a reduction in right ventricular and atrial size and by bulging of the atrial septum into the left atrium. To assess PFO, semi-quantification of the right to left shunt (RLS) was performed. On TEE the evaluation was based on counting the number of micro bubbles (MBs) moving from the right atrium to the left atrium through the PFO after the Valsalva maneuver, within the first three cardiac cycles. The studies were considered positive for PFO when at least one micro bubble was observed in the left atrium. The severity of the shunt was quantified as mild (<10 micro bubbles), moderate (10–20 micro bubbles), or severe (>20). If the quantitative results varied between the first and second study the largest number of MBs decided about the shunt size.

### Statistical analysis

The SPSS 13.0 software package was used for statistical analysis. Quantitative data were expressed as the mean ± standard deviation, qualitative data as the percentage. The performance indexes used were sensitivity, specificity, predictive positive value (PPV), negative predictive value (NPV), and k concordance index, which express the agreement proportion beyond chance.

Sensitivity was defined as:

$$\mathrm{sentivity}=\frac{\mathrm{number}\;\mathrm{of}\;\mathrm{True}\;\mathrm{Positives}}{\mathrm{number}\;\mathrm{of}\;\mathrm{True}\;\mathrm{Positives}\;+\;\mathrm{number}\;\mathrm{of}\;\mathrm{False}\;\mathrm{Negatives}}$$

Specificity was defined as:

$$\mathrm{specificity}=\frac{\mathrm{number}\;\mathrm{of}\;\mathrm{True}\;\mathrm{Negatives}}{\mathrm{number}\;\mathrm{of}\;\mathrm{True}\;\mathrm{Negatives}\;+\;\mathrm{number}\;\mathrm{of}\;\mathrm{False}\;\mathrm{Positives}}$$

## Results

Using both TCD and TEE, we detected a right-to-left shunt in 220 patients (52.3%) and no-shunts in 159 patients (37.9%). In 20 (4.8%) patients TEE did not show the passage of contrast which was later detected by TCD. In 21 (5.0%) patients only TEE revealed a PFO. The feasibility of both methods was 100%. Shunts confirmed by both methods (TCD and TEE) were found in: 78.4% of patients with cryptogenic stroke, in 48.6% of patients with TIA, and only in 10.4% of patients with migraine without aura (Table 2). Considering TEE as the gold standard technique, TCD was able to identify 200 (91.3%) of the 241 patients with PFO. TCD showed a NPV of 89%, PPV of 98%, sensitivity of 95%, and specificity of 92%. TCD showed a high concordance (k = 0.89) with TEE in PFO recognition (Table 3). Shunt grading in both TCD and TEE methods was classified with the same score in 70.5% of cases, and it was the highest in the large shunt (Table 4).

**Table 2:** Shunt grading using both transcranial doppler ultrasonography and transesophageal echocardiography

**SHUNT**	**TEE (N= 220 pts – TEE and TCD positive)**	**TCD (N= 220 pts – TEE and TCD positive)**	**% of the same grading using both methods**
**Small**	56 (25.5%)	70 (31.8%)	62.1
**Medium**	123 (55.9%)	119 (54.1%)	68.3
**Large**	41 (18.6%)	31 (14.1%)	83.2

*TCD* Transcranial Doppler Ultrasonography, *TEE* Transesophageal Echocardiography.

**Table 3:** Identification of persistent for amen ovale using both transcranial color doppler and transesophageal echocardiography

**Clinical characteristics of patients**	**Identification of PFO (both TCD and TEE)**	
	**Patients (n = 420)**	**Patients (n = 220)**
TIA	218 (51.9%)	106/218 (48.6%)
Migraine	67 (15.6%)	7/67 (10.4%)
Migraine with aura	50 (11.9%)	38/50 (76.0%)
Cryptogenic stroke	88 (20.9%)	69/88 (78.4%)

*PFO* Patent Foramen Ovale, *TCD* Transcranial Doppler Ultrasonography, *TEE* Transesophageal Echocardiography, *TIA* Transient Ischaemic Attack.

**Table 4:** Sensitivity, specificity, negative predictive value, and positive predictive value of transcranial doppler ultrasonography in comparison with transesophageal echocardiography in pfo detection

**Technique**	**PFO (%)**	**PPV (%)**	**NPV (%)**	**Sensitivity (%)**	**Specificity (%)**
Contrast-enhanced TCD	90	98	89	95	92

NPV: Negative Predictive Value, PPV: Negative Predictive Value TCD: Transcranial Doppler Ultrasonography, TEE: Transesophageal Echocardiography, PFO: Patent Foramen Ovale.

## Discussion

Patent foramen ovale is a hemodynamically trivial interatrial communication that is present in about 25% of the adult population. However, the clinical significance of PFO in relation to cerebrovascular events is still debated, identification and assessment of this abnormality are nowadays a routine diagnostic procedure [6,9-12]. The search for PFO is becoming more and more common in young subjects suffering from migraine, TIA or cryptogenic stroke [12-17]. At the moment there is no clearly recognized standard protocol for performing both TEE and TCD studies to identify PFO. For the first time, based on the available literature, we developed a standardized exercise protocol in both TEE and TCD studies.

Contrast transesophageal echocardiography is the gold standard for the diagnosis of PFO, regardless of the fact that this procedure is semi-invasive [18-24]. Therefore, transcranial Doppler ultrasonography seems to be an attractive alternative to TEE in the recognition of PFO, because it is a cheap and noninvasive examination [25-29].

The aim of this study was to evaluate the feasibility of transcranial Doppler and its diagnostic sensitivity compared with transesophageal echocardiography. Although the diagnostic power of TCD has been reported, only a few studies comparing both techniques (TCD and TEE) have been published [5,30-33]. In our study TCD had a good sensitivity and specificity. In our opinion, in contrast to other investigators, TCD and TEE are complementary for PFO diagnosis, but TCD should be recommended as the first choice for screening because of its simplicity, non-invasive character, low cost, and high feasibility. Contrary to other researchers, our results and clinical experience show that both methods are necessary and complementary to recognize a significant foramen ovale.

The sensitivity and specificity of TCD for shunt detection vary in different studies according to both protocol and diagnostic criteria [25-33]. In our study TCD had 89% NPV, 98% PPV, 95% sensitivity, and 92% specificity. In the Caputi study a general concordance of up to 90% between TEE and TCD was found. TCD sensitivity and specificity were 96.8% and 78.4%, respectively [25].

Several studies have suggested that the low specificity of TCD may be due to the fact that TCD is able to detect both cardiac and pulmonary shunts [5]. The specificity for a PFO shunt may be increased by a defined time of micro bubble appearance. The passage time from the antecubital vein injection site to the middle cerebral artery (MCA) through an intracardiac shunt is about 11 seconds, and it is about 14 seconds in the case of pulmonary passage. The overlap interval between intrapulmonary and cardiac passage level must be taken into account [33].

In our study only in 4.8% of patients TEE did not show the passage of contrast which was later detected by TCD. The meaning of a shunt detected only during TCD is not clearly understood, but a very small intracardiac shunt, not detected by TEE, cannot be excluded. Furthermore the Valsalva maneuver may be less effective during TEE than during TCD, because of esophageal intubation. It is crucial to perform the Valsalva maneuver properly. Contrary to other investigators, in our material, the patients had been trained in performing the Valsalva maneuver before study.

Recent studies as well as our results (95% sensitivity, and 92% specificity of TCD), show a good concordance between TCD and TEE with sensitivity ranging from 70% to 100% and specificity >95% [27,31,32].

Both techniques have class IIA recommendations for shunt detection [33].

Taking into account that TCD and TEE have similar sensitivity and specificity in shunt detection TCD should be recommended as the first choice for screening because of its low cost, and high feasibility. TCD is also a noninvasive exam - easy to perform and repeat, if necessary. TEE should be limited to the patients scheduled for transcatheter PFO closure, patients with high-risk PFO with recurrent stroke, and patients with ASA or large shunt detected on TCD.

## Conclusion

TCD has a relatively good sensitivity and specificity. TCD and TEE are complementary for PFO diagnosis, but TCD should be recommended as the first choice for screening because of its simplicity, non-invasive character, low cost, and high feasibility.

## Abbreviations

MBs: Micro bubbles; MCA: Middle cerebral artery; NPV: Negative predictive value; PFO: Persistent foramen ovale; PPV: Positive predictive value; RLS: Right to left shunt; TCD: Transcranial color doppler; TEE: Transesophageal echocardiography; TIA: Transient ischaemic attack; TTE: Transthoracic echocardiography.

## Competing interests

The authors declare that they have no competing interests.

## Authors’ contributions

MK: contributed to study conception and design, acquisition of data, analysis and interpretation of data, drafting the manuscript, revising it critically for important intellectual content. MO: contributed to study conception and design, helped to draft the manuscript, revising it critically. TP: contributed to study conception and design, acquisition of data, analysis and interpretation of data, drafting the manuscript, revising it critically for important intellectual content, gave final approval of the version to be published. JP: contributed to study conception and design, analysis and interpretation of data. JS: analysis and interpretation of data, drafting the manuscript. BS: contributed to study conception and design, acquisition of data, analysis and interpretation of data, drafting the manuscript. RB: contributed in revising critically for important intellectual content. AKZ: analysis and interpretation of data, revising it critically for important intellectual content. LTP: contributed in acquisition of data, analysis and interpretation of data, drafting the manuscript, revising it critically for important intellectual content. PP: contributed to study conception and design, revising it critically for important intellectual content, gave final approval of the version to be published. All author’s read approved the final manuscript.
